# Newborn Screening in Developing Countries: The Need of the Hour

**DOI:** 10.7759/cureus.59572

**Published:** 2024-05-03

**Authors:** Sarika Gaikwad, Shubhangi Ganvir, Punam Uke

**Affiliations:** 1 Department of Pediatrics, Jawaharlal Nehru Medical College, Datta Meghe Institute of Higher Education and Research, Wardha, IND; 2 Department of Pediatrics, Grant Medical College and Sir Jamshedjee Jeejeebhoy Group of Hospitals, Mumbai, IND

**Keywords:** mental disability., cost effectiveness, developing countries, dried blood spot, newborn screening program

## Abstract

Screening newborns is recognized as an important health policy. It is cost-effective and is implemented as a national health program in most developed countries. Though births in developing countries contribute to more than half of the total births globally, newborn screening (NBS) is not yet implemented in most developing countries. If not diagnosed and treated timely, some of these infants will contribute to neonatal mortality. In contrast, others will have long-term sequelae like developmental delay, learning disabilities, behavioral abnormalities, and backward academic performance in the future. In addition, the diagnosis, management, and treatment of these conditions also carry a significant financial as well as emotional burden on the family. An NBS program can be the most rational and effective way to prevent such morbidities and mortalities. NBS in developing countries competes with other health issues such as the control of infectious diseases, vaccinations, and poor nutrition. Also, lack of government support, poor economy, inadequate public health education, lack of awareness among health care workers, early discharge from hospital, and many births out of hospital are the significant obstacles in the countries that lack total coverage. It is high time now to change our attitude; our focus should be not only on the reduction of mortality and infectious morbidity but also on reducing disabilities with the introduction of screening for newborns. Integrating NBS with the national healthcare system is crucial for successful implementation in developing countries. Integration should also include a payment scheme to reduce the economic burden on families. In recent years, many developing countries have started implementing pilot projects as a step toward the national program of screening newborns.

## Introduction and background

One of the most critical public health initiatives of this decade is screening newborns. Newborn screening (NBS) tests the dried blood spot (DBS) of all newborns for certain diseases that are not noticed at birth but, if not diagnosed and treated timely, can lead to serious disability, developmental delay, and even death. These infants seem to be healthy and have a negative family history in most cases. It is being adopted in the vast majority of developed countries. However, in most developing countries, it is still in its development phase and has yet to enter the implementation stage as a national program. In Bangalore, India, the first NBS program was implemented in 1980, screening 125,000 infants for a range of metabolic abnormalities. This showed that the main causes of mental retardation in our community include conditions like glycinemia, hyperphenylalaninemia, tyrosinemia, and maple syrup urine disease. Infants are also investigated for galactosemia, cystic fibrosis (CF), biotinidase deficiency, glucose-6-phosphate dehydrogenase (G6PD) deficiency, congenital adrenal hyperplasia (CAH), and amino acid abnormalities. The findings show that congenital hypothyroidism (CH) is highly prevalent [1]. Worldwide, CH occurs in 1:3,000 people, with a higher prevalence rate in iodine-deficient areas at around 1:900 [2]. Considering the total annual births in developing countries in Asia, about 22,200, and even more due to limited documentation potential, new cases of CH are born every year. However, only 10% of cases are being screened [3]. Screening for CH may prevent a significant proportion of children from developing intellectual disability. An estimated 50.9 inborn errors of metabolism (IEMs) are present for every 100,000 live births worldwide [4]. Based on statistics from Indian pediatrics, 1 in 2,497 infants have IEMs [5], 2.1 out of 1,000 have CH [6], and 2%-7.8% have G6PD deficiency [7].

In India, three public screening programs were initiated: the Chandigarh Program, the Goa Program, and the Kerala Program. The fatty acid oxidation disorders, organic acid disorders, and amino acid disorders, which tandem mass spectrometry (MSMS) screens, are excluded from these screening programs due to resource limitations, high cost, and shortage of experts. The Chandigarh Program was launched by the Union Territory of Chandigarh, India, in 2007 to investigate the occurrence of three disorders (CH, CAH, and G6PD deficiency). The Goa 1.0 NBS Program was started in 2008 and continued until 2013 with the goal of improving neonatal care in the region. It was believed that implementing NBS could help improve the statistics related to neonatal care in Goa. The program encompassed a comprehensive panel of over 50 disorders. The Goa 2.0 NBS Program was launched in August 2018, which focuses on screening all neonates from government hospitals for six disorders, namely CH, CAH, G6PD, galactosemia, galactose-1-phosphate uridyl transferase (GALT), biotinidase, and CF [8]. The Kerala Program, which was launched in 2012, screens for four main disorders (CH, CAH, G6PD, and GALT) for all births from government hospitals in 2018 [9].

To prevent the children from long-term disabilities and mortality, the newborn must undergo screening. The goal of NBS is to detect the disorders as early as possible so that prompt action can be taken to avoid major long-term health effects. NBS is particularly important in the Asia-Pacific region, where almost half of the world's births occur (68.5 million of total 136.7 million births). China, India, Indonesia, Bangladesh, and Pakistan account for 85% of global births [10]. Despite all these obstacles, some developing countries have understood the importance of NBS and have started implementing pilot projects for NBS as a step toward it.

## Review

Diseases that can be screened by NBS

The foundation of NBS was laid in the 1960s by Robert Guthrie, a microbiologist whose child was suspected of having phenylketonuria (PKU) [11]. This testing procedure allowed a majority of newborns to be screened for PKU in a short duration. Studies have shown that a diet low in phenylalanine helps prevent mental retardation if started soon after birth before the appearance of symptoms [12]. Subsequently, many countries have understood the importance of NBS for PKU. As part of the NBS panel, an immunoassay for thyroid-stimulating hormone (TSH) and thyroxine screening was developed for CH following PKU diagnosis. Screening for CF has gained importance since there is a lot of evidence of better pulmonary outcomes, improved nutrition, and increased long-term survival if the infant is screened at birth. There is similar evidence for other disorders that can be diagnosed using MSMS. When considering which medical conditions to include in the NBS panel, it is not practical to add all possible conditions. Hence, to decide which conditions are appropriate for the NBS panel, Wilson and Junger [13] established criteria in 1968, which have been summarized in Table 1.

**Table 1:** Wilson and Jungner's criteria for NBS NBS: newborn screening

Wilson and Jungner's criteria for NBS	
1.	The illness needs to be a serious medical problem.
2.	Patients with recognized diseases have to receive an accepted course of therapy.
3.	There should be facilities for patient diagnosis and therapy.
4.	A distinct latent or early symptomatic stage must present.
5.	An appropriate test or examination should be available.
6.	The test should be acceptable.
7.	It is important to have an adequate knowledge of the natural history, progress, and latent disease.
8.	The diagnosis and treatment should be cost-effective.
9.	A common policy regarding who should be treated as a patient is required.
10.	Finding cases should not be a "once and for all" endeavor but rather an ongoing one.

NBS sample

Cord blood samples were initially used in areas where discharge is given early after delivery. However, the chances of maternal contamination are high in cord samples. Also, only some of the disorders like CH can be tested with cord blood samples [14]. In addition, it has a very limited value in detecting disorders by MSMS. It has been evident from many studies that better results can be obtained from the heel prick sample [15]. The heel prick sample is collected on a high-quality cotton fiber-based paper known as a Guthrie card. The sample is allowed to dry adequately for a few hours before being sent to the central NBS laboratory. It only takes a few drops of blood to finish the NBS panel. The timing of sample collection is crucial because some of the metabolites and hormones vary significantly over time and thus can lead to false results. Some markers decrease, while some increase with age. Hence, sample timing is a compromise, and the current recommendation of sampling is at 48-72 hours of life [16]. Some other factors, such as birth weight, prematurity, hyperbilirubinemia, transfusion, type of feeding, and parenteral nutrition, can also influence the NBS results and hence need to be considered before interpreting the results. Hence, this information needs to be mentioned on the NBS card. Heat, humidity, and transportation delays can cause degradation of some markers and thus can lead to false-negative reports. Urine samples, as a screening tool for NBS for PKU, have been used for a long time in stems using ferric chloride reagent [17]. However, it was later discovered that it is a relatively insensitive test for screening PKU. This test has recently gained importance since some of the IEMs that cannot be detected with blood samples can be diagnosed by urine tests using MSMS [18]. NBS has dramatically evolved from a simple bacterial inhibition assay to complex methodologies like gas chromatography-mass spectrophotometry, MSMS, microarray, and next-generation sequencing (NGS). This NBS panel now goes beyond the analysis of blood spots. The usage of genetic testing in NBS has increased recently due to the tremendous advancements in this field. In general, diseases with unusual clinical presentations, complex phenotypes, and multigene pathogenicity can be treated with technologies like Sanger sequencing, quantitative polymerase chain reaction (qPCR), and NGS sequencing, which includes panel sequencing, whole-exome sequencing, and whole-genome sequencing [19]. When compared to genetic testing, NGS is a highly efficient method as it simultaneously sequences huge panels of genes in a single assay.

PKU and Hyperphenylalaninemia

PKU affects around 0.45 million people globally, with an estimated prevalence of one in 23,930 live births [20]. PKU is characterized by an excessive level of phenylalanine in the blood due to mutations in the phenylalanine hydroxylase gene. The DBS level of phenylalanine can be as high as >200 micromol/L and increase the phenylalanine-to-tyrosine ratio. The high level is detrimental to early brain development and can lead to developmental delay and intellectual disability. Dietary restriction of phenylalanine can prevent the long-term consequences of PKU if initiated from the early newborn period [21]. Hence, early NBS and prompt initiation of treatment can prevent infants from long-term undesirable consequences resulting from PKU.

Congenital Hypothyroidism

One of the avoidable causes of intellectual disability is CH. CH has a birth prevalence of 1:2,750 [22]. The most frequent cause of hypothyroidism in the world is iodine deficiency. TSH, or thyroxine-binding globulin values, and thyroxine, in different combinations, help detect CH by NBS using immunoassay [23]. Most countries perform a single TSH test since it is relatively simple and the chance of false positivity is low. However, central hypothyroidism cannot be detected with this method [24]. Furthermore, it may lead to false-negative screening results in premature and low-birth-weight infants [25]. Some of the contrast agents and iodine-containing disinfectants can lead to a false-positive result in the screening test [26]. Hence, positive screening results need to be followed up further with thyroid function tests and thyroid scans.

Galactosemia

The overall incidence of galactosemia varies by race and ethnicity. It is the highest among Caucasians and ranges between 1:16,000 and 1:44,000 among infants in the United Kingdom and Ireland. Galactosemia is an autosomal recessive disorder produced due to GALT deficiency. The majority of NBS systems use enzymatic assays to determine "galactose metabolites," such as galactose and galactose-1-phosphate, for the detection of galactosemia [27]. Galactosemia, if not treated promptly, can lead to life-threatening complications like failure to thrive, *Escherichia coli* sepsis, and hepatocellular damage. Long-term consequences include developmental delay and speech problems (dysarthria and speech apraxia). Thus, early screening of galactosemia can prevent neonatal mortality as well as morbidity. However, most of the cases with typical symptoms can be diagnosed clinically regardless of NBS [28].

Cystic Fibrosis

The reason CF occurs is due to the mutant CF transmembrane conductance regulator (CFTR) gene, which results in altered chloride transport and leads to severe damage to the lungs, digestive system, and other organs. Immunoassay measurement of immunoreactive trypsin (IRT) is widely used in NBS programs [29]. However, it is a relatively unstable marker, and results are affected due to improper storage or delay in the analysis of the DBS sample. Detection of the CFTR gene and disease-causing mutations provides superior performance. Hence, most of the NBS programs have started using the IRT/CFTR mutation protocol [30]. The gold standard method for diagnosing CF is through sweat chloride testing, which measures chloride levels >60 mmol/L [31]. Early commencement of physiotherapy and antibiotic treatment significantly improves the long-term prognosis.

Inborn Errors of Metabolism

With the development of MSMS by Millington et al., it has become possible to diagnose many IEMs involving fatty acid and organic acid metabolism simultaneously [32]. DBS is introduced into the electrospray ionization source without chromatography with a run time of less than two minutes [33]. Medium-chain acyl-Co-dehydrogenase deficiency, a disorder of fatty acid oxidation, if not diagnosed early, can lead to sudden unexplained infant death. Table 2 summarizes other IEMs detected by MSMS. With MSMS, multiple disorders can be screened at once through multiplex tests.

**Table 2:** Enzyme deficiencies and disorders detected using MSMS by NBS ^a^Milder forms may be missed ^b^Missed cases are likely ^c^Uncertain clinical significance, not included in some programs MSMS: tandem mass spectrometry; NBS: newborn screening

Amino acid disorders
Homocystinuria
Citrullinemia type I
Maple syrup urine disease^a^
Argininemia
Argininosuccinic aciduria
Citrin deficiency^b^
Phenylketonuria and pterin defects
Tyrosinemia type I^b^
Tyrosinemia type II
Fatty acid oxidation disorders
Carnitine uptake defect
Carnitine palmitoyl transferase I deficiency
Carnitine palmitoyl transferase II deficiency
Carnitine:acylcarnitine translocase deficiency
Long-chain hydroxy acyl-CoA dehydrogenase/trifunctional protein deficiency
Medium-chain acyl-CoA dehydrogenase deficiency
Multiple acyl-CoA dehydrogenase deficiency
Short-chain hydroxyacyl-CoA dehydrogenase deficiency^c^
Short-chain acyl-CoA dehydrogenase deficiency
Very long-chain acyl-CoA dehydrogenase deficiency
Organic acid disorders
3-methylcrotonyl-CoA carboxylase deficiency^c^
3-hydroxy-3-methylglutaryl-CoA lyase deficiency
2-methylbutyryl-CoA dehydrogenase deficiency
3-methylglutaconic aciduria type I deficiency
Biotinidase deficiency^a^
Beta-ketothiolase deficiency
Holocarboxylase deficiency
Isobutyryl-CoA dehydrogenase deficiency​​​​​​​^c^
Isovaleric acidemia
Propionic acidemia
Methylmalonic acidemias
Isobutyryl-CoA dehydrogenase deficiency^c^
Holocarboxylase deficiency
Glutaric aciduria type I deficiency
Cobalamin C disease

Congenital Adrenal Hyperplasia

The prevalence of CAH in India is reported to be one in 5,762 infants, as per NBS data [34]. CAH is caused by defects in steroid metabolism, resulting in less production of mineralocorticoid and glucocorticoid and excess production of androgen [35]. In some cases, infants may present with catastrophic salt-wasting events, and female neonates may develop male characteristics. NBS testing involves immunoassay measurement of 17-hydroxyprogesterone (17-OHP). However, classical CAH is diagnosed clinically due to masculinization of genitalia, thus diminishing the value of NBS for diagnosis of CAH. The majority of CAH results from 21-hydroxylase deficiency, while a few are caused by 11β-hydroxylase, both resulting in an increased 17-OHP level [36].

Hemoglobin Disorders

Hemoglobin disorders have been designated a severe issue for public health by the WHO. The carrier frequency of beta thalassemia varies from 1% to 17% in developing nations like India [37]. Sickle cell disease is a significant medical illness in some developing nations like Nigeria, India, and the Democratic Republic of Congo. Also, it has been demonstrated from many studies in several countries that early diagnosis and timely adequate care can prevent lethal complications in sickle cell disease. NBS helps in the identification of infants with hemoglobin disorders early, even before the onset of symptoms, thus preventing resulting complications.

Glucose-6-Phosphate Dehydrogenase Deficiency

The worldwide prevalence of G6PD deficiency is more than 400 million, specifically high in the tropics and subtropics, areas of malarial endemicity [38]. Males are normally hemizygous for the gene, while females can have normal genes or heterozygous, homozygous, or compound heterozygous for the G6PD gene. According to Vulliamy et al., subtropical areas have extremely high frequencies of the G6PD-deficiency allele [39]. In most developing countries, accessibility to phototherapy, as well as exchange transfusion, is often limited. Also, despite simple and accessible treatment, even in developed countries, roughly 50% of infants with kernicterus will pass away, and 6.6% of G6PD-deficient infants will get kernicterus [40]. Considering the high gene pool in the population, universal screening is essential. Thus, screening for G6PD can triage neonates to prevent kernicterus and long-term neurodevelopmental complications.

Inborn Errors of Immunity

Primary immunodeficiency diseases are a category of around 500 monogenic disorders that, if undiagnosed, can result in severe morbidity and death. Patients with primary immunodeficiency are more susceptible to recurring life-threatening infections, development of autoimmunity, and, at times, malignancy. The cumulative incidence and prevalence of IEIs are one in 4,000 and one in 1,000, respectively [41]. Severe combined immunodeficiency is a prototype for NBS that has been developed and is routinely performed in most developed countries. Quantitative estimation of T-cell receptor excision circles by qPCR is the primary tool currently utilized for NBS [42].

Screening for Newborn Hearing

Significant hearing loss affects one to two neonates per 1,000 healthy newborns, while the percentage increases by 2%-3% in neonatal intensive care unit admissions. Around 30% of people are affected by congenital hearing loss. Congenital or acquired hearing loss can result in several behavioral, social, and emotional problems, with major delays in language and speech development and academic achievement. At three years of age, a child who receives early assistance at birth has a vocabulary of 300-700 words. If they receive intervention at six months, their vocabulary decreases to 150-300 words. However, if they receive intervention at two years, their vocabulary drastically decreases to just 0-50 words. This emphasizes the importance of early detection and correction of hearing loss in children [43]. Infants can be checked in two stages: those who fail or refer to the otoacoustic emissions should be screened again with auditory brainstem response.

Screening for Congenital Heart Disease

There are no consensus guidelines for screening congenital heart disease (CHD). The American Academy of Pediatrics and American Heart Association recommend that NBS should be done after 24 hours and before the discharge of the neonate from the hospital. Neonates with a saturation of less than 90% should undergo immediate ECHO and those with a saturation of less than 95% should be monitored closely and reassessed [44]. A practical, safe, and noninvasive method for detecting CHD is pulse oximetry. Critical heart disease can be detected with a 73% sensitivity and a 99.9% specificity [45].

Hindrances in the implication of NBS

The following are some of the hindrances to the introduction of NBS in underdeveloped nations.

Population: Of 113 million births worldwide, 66.9 million (almost half) are from Asia. About 80% of births are from five countries, namely India, China, Pakistan, Indonesia, and Bangladesh. In these countries, more than two-thirds of the population is still living in rural areas, where they struggle for basic living amenities and suffer from malnutrition and many infectious diseases.

Active participation in all sectors of health: All members of the health care delivery system, including community health workers, nurses, geneticists, obstetricians, neonatologists, and pediatrics and hospital administration, as well as policymakers, need to be empowered and sensitized regarding the importance of NBS.

Inadequate knowledge of healthcare providers: Most healthcare providers in the developing world lack sufficient knowledge regarding NBS. Also, once an infant has been verified to be positive for a specific illness, there are relatively few specialists to whom referral can be made.

Family: Parents, as well as other family members, are often unaware of the importance of NBS at the beginning of the program. Early discharge from the hospital also contributes to negative consent from the parents since they decide not to screen them after being discharged. Money is also another major issue for family members.

Cost-effectiveness of NBS: Even though NBS is seen as a costly therapy, its advantages surpass its costs because it greatly lowers morbidity and mortality. Further, if these diseases are not detected and treated early, the cost of diagnosis and treatment may end up being significantly higher. Also, if early detection and management of these diseases are not made, the disability, be it mental or physical, will pose significant economic, social, and emotional burdens to the individual. The cost of these burdens is much higher than the cost of NBS.

Recommendations

All developing countries should be encouraged to develop policies and create a comprehensive national program for neonatal screening. NBS should focus on specific common diseases in a particular belt. NBS programs need to be integrated into the national healthcare delivery system. Full government prioritization is very essential for the smooth implementation and success of NBS programs. There should be full or partial government financing. Pilot studies should be encouraged. The countries in the budding phase of implementing NBS will require advice from developed nations with more advanced systems. Hence, nations start cooperating by exchanging knowledge, resources, and skills. It is necessary to establish and carry out training curriculums for NBS. Since it is not possible to screen all disorders as screened by developed countries with more than 90% NBS coverage, each country should focus on screening specific disorders common in a particular country. In India, it is recommended to include screening programs for treatable disorders like CH, CAH, G6PD, galactosemia (GALT), biotinidase, CF, and hemoglobinopathy variations such as sickle cell disease in target populations.

## Conclusions

Thus, neonates born with common disorders like CH, CHD, hearing deafness, and G6PD deficiency in developing countries are still waiting for universal screening. NBS is a set of assessments given to newborns to detect potentially serious but clinically occult illnesses that need prompt attention. Every newborn has the right to be screened for diseases that are avoidable and can be found using basic NBS methods so that they can have a better quality of life with early interventions. It is high time now that our focus should not be just on the reduction of mortality and infectious morbidity but also on reducing disabilities with the successful implementation of NBS.
